# Corneal Nerve Assessment by Aesthesiometry: History, Advancements, and Future Directions

**DOI:** 10.3390/vision8020034

**Published:** 2024-05-12

**Authors:** Jordan R. Crabtree, Shadia Tannir, Khoa Tran, Charline S. Boente, Asim Ali, Gregory H. Borschel

**Affiliations:** 1 Department of Surgery, Indiana University School of Medicine, Indianapolis, IN 46202, USA; jrcrabtr@iu.edu (J.R.C.);; 2 Department of Ophthalmology, Indiana University School of Medicine, Indianapolis, IN 46202, USA; 3 Department of Ophthalmology and Vision Sciences, Hospital for Sick Children, Toronto, ON M5G 1X8, Canada; 4 Department of Ophthalmology and Vision Sciences, University of Toronto, Toronto, ON M5S 1A1, Canada

**Keywords:** cornea, corneal sensitivity measurement, aesthesiometry, Cochet–Bonnet, neurotrophic keratopathy, neurotrophic keratitis

## Abstract

The measurement of corneal sensation allows clinicians to assess the status of corneal innervation and serves as a crucial indicator of corneal disease and eye health. Many devices are available to assess corneal sensation, including the Cochet–Bonnet aesthesiometer, the Belmonte Aesthesiometer, the Swiss Liquid Jet Aesthesiometer, and the newly introduced Corneal Esthesiometer Brill. Increasing the clinical use of in vivo confocal microscopy and optical coherence tomography will allow for greater insight into the diagnosis, classification, and monitoring of ocular surface diseases such as neurotrophic keratopathy; however, formal esthesiometric measurement remains necessary to assess the functional status of corneal nerves. These aesthesiometers vary widely in their mode of corneal stimulus generation and their relative accessibility, precision, and ease of clinical use. The development of future devices to optimize these characteristics, as well as further comparative studies between device types should enable more accurate and precise diagnosis and treatment of corneal innervation deficits. The purpose of this narrative review is to describe the advancements in the use of aesthesiometers since their introduction to clinical practice, compare currently available devices for assessing corneal innervation and their relative limitations, and discuss how the assessment of corneal innervation is crucial to understanding and treating pathologies of the ocular surface.

## 1. The Cornea and Neurotrophic Keratopathy:

The cornea is the most densely innervated tissue of the human body, 400 times more densely innervated than the skin [1]. Adequate corneal innervation is vital to many aspects of normal ocular surface function, including maintenance of the tear film, perception of foreign bodies, recognition of noxious stimuli, and neurotrophic influences on the corneal epithelium [2,3,4].

Impairment of the protective and neurotrophic functions of the cornea leads to an inability to maintain corneal integrity, as well as inadequate recovery after even minor corneal abrasion or injury. The condition resulting from insufficient corneal innervation jeopardizing epithelial integrity is termed neurotrophic keratopathy (NK). Though the molecular basis of NK is still under study, evidence suggests it may ultimately be due to the loss of innervation-dependent paracrine signaling interactions in the corneal limbus [4,5]. In the basal limbal epithelium, axons adjacent to limbal stem cells (LSCs) are typically responsible for stimulating LSC activity, leading to LSC differentiation into transient amplifying cells (TACs). In the absence of pathology, these TACs migrate toward the central cornea, differentiate further into epithelial cells, and mediate corneal homeostasis or epithelial repletion after injury [4]. When these axons are absent or non-functional, such as in NK, a variety of consequences secondary to impaired trophic activity of the epithelium can arise. These include infection, corneal and stromal scarring, perforation, ulceration, and potentially blindness [6].

In addition to direct trophic stimulation of LSCs by nerves, evidence has shown that neuroimmune interactions with populations of innate corneal immune cells likely regulate a portion of epithelial maintenance through the release of various neurotrophic substances [7,8,9]. These interactions likely play a role in the pathogenesis and progression of NK, and corneal nerve disruption has been shown to be associated with reductions in two neurotransmitters implicated in corneal epithelial healing—substance P and acetylcholine [8,10]. Additional factors from non-neuronal sources suggested to regulate corneal epithelial healing include ciliary neurotrophic factor (CNTF), transforming growth factor-β (TGF-β), platelet-derived growth factors (PDGFs), and nerve growth factor (NGF) [4]. Only NGF has been shown to be effective in clinical studies, and topical recombinant human NGF is currently the only FDA-approved treatment for NK [11].

NK is traditionally staged according to the Mackie classification system, which defines three stages based upon epithelial integrity, though additional criteria have been suggested in recent years to include imaging parameters obtained through in vivo confocal microscopy (IVCM) and optical coherence tomography (OCT) [12]. Stage 1 NK is defined by punctate corneal epithelial fluorescein staining, increased mucous viscosity, and decreased tear breakup time. Stage 2 NK includes epithelial defects typically surrounded by a rim of loose epithelium or rolled edges, as well as stromal swelling and occasionally inflammatory anterior chamber activity. Stage 3 NK is defined by stromal lysis or melting, which can lead to perforation [13]. Subjective complaints in the early stages of NK often include eye redness, dryness, or fatigue, as well as visual disturbances such as sensitivity to light, blurred vision, and reduced visual acuity rather than loss of sensation [14].

Neurotrophic keratopathy’s progressive nature is due to a variety of sequelae stemming from nerve dysfunction. Corneal damage is compounded by the loss of protective ocular reflexes. With suppression of the blink reflex, patients sustain repetitive epithelial microtraumas, which lead to eventual corneal and stromal scarring, affecting corneal clarity [4,15]. Impairments in lacrimation lead to reduced clearance of irritants and poor distribution of tears and factors responsible for maintaining epithelial integrity, compromising ocular surface health. NK has a variety of genetic and acquired etiologies, all of which lead to impairment or absence of the afferent pathways innervating the cornea. These include diabetes, central nervous system tumors or tumor resections, ocular surgery, herpetic infections, hindbrain developmental conditions, and others.

Accurate assessment of corneal sensation represents an important aspect of the diagnosis, assessment, and management of NK. If diagnosed early, clinicians can prevent the otherwise inevitable progressive deterioration of the ocular surface that is the hallmark of the condition. Further, using diagnostic devices that can accurately and precisely measure the extent of innervation, clinicians can track the progress of their patients with interventions designed to improve the level of innervation, escalating to the next therapeutic option when warranted. In patients with particularly impaired sensation, this may be in the form of escalation from symptomatic management to dedicated therapeutics, or in severe cases, surgical intervention by corneal neurotization.

## 2. Corneal Nerves and Their Stimulation

From the ophthalmic division of the trigeminal ganglion, sensory nerve fibers travel in the nasociliary nerve and long ciliary nerve branches before contacting the cornea. Dividing further, they form a large network of overlapping sensory-receptive fields that provide extreme sensitivity to stimuli, but poor stimulus localization [16].

There are three major nociceptor types responsible for corneal sensation within these receptive fields, and nerve impulses are conducted through a combination of thin, myelinated Aδ-type fibers, or slower, unmyelinated C-type fibers. Approximately 20% of nociceptors are mechanoreceptors responsible for the transmission of acute, sharp pain from mechanical contact at the cornea that is near the amount required to cause damage to the corneal epithelium. This takes place exclusively through the fast-transmitting Aδ-type nerve fibers. Polymodal nociceptors, the most abundant nociceptor type, also communicate sharp and sustained pain from mechanical contact to the cornea, and are additionally activated by irritant chemical stimuli, endogenous inflammatory mediators, and extreme temperatures. These comprise approximately 70% of nociceptors, and the majority communicate through the slower C-type fibers, with few utilizing Aδ fibers. In contrast to mechanoreceptors—which are only able to communicate limited information about a stimulus’ intensity—polymodal nociceptors transmit both intensity and duration of an irritant stimulus. Finally, both Aδ- and C-type fibers encode information from “cold receptors”, which respond to corneal cooling and evaporation of the tear film. These compose the final approximately 10% of nociceptors [16,17,18]. Due to its extremely dense plexus of nerves and diverse nociceptor characteristics, the ability of the cornea to detect a variety of insults has been leveraged by numerous devices aimed at assessing corneal sensory function.

Currently employed corneal aesthesiometers vary in their method of stimulus generation, and subsequently generate different results in their assessment of sensation. The simplest and currently recognized gold standard, the Cochet–Bonnet aesthesiometer, delivers mechanical stimulation alone with an adjustable length of nylon filament. Non-contact aesthesiometers measure the amount of airflow required to trigger a patient response. The Swiss Liquid Jet Aesthesiometer uses a jet of fluid isotonic to the corneal surface to determine the total volume of liquid used to generate a corneal response. Most recently developed, the Corneal Esthesiometer Brill delivers varying pressure air pulses to the corneal surface to generate a response. Because these devices use different methods to generate stimuli, measure responses in different units, and apply their forces over different areas, challenges arise in comparing the amount of pressure applied to the cornea by each.

## 3. Etiologies of Corneal Hypoesthesia

A variety of conditions, both systemic and isolated to the eye, can result in decreased corneal sensation and, subsequently, NK, and therefore warrant evaluation by corneal aesthesiometry (Table 1). Corneal innervation is intricately tied to the health of the corneal epithelium, and adequate assessment of these nerves is important for timely treatment and the prevention of potentially vision-threatening complications.

Ocular-specific causes of decreased corneal sensation include infectious, congenital, idiopathic, and even iatrogenic etiologies. Any condition causing impairment of the trigeminal nerve can, in turn, lead to impairment of corneal innervation and subsequent epithelial defects. The most common causes of corneal anesthesia include keratoconjunctivitis due to viral infections by herpes simplex and herpes zoster, trauma such as chemical burns and mechanical injuries, CNS tumors, and corneal or other ocular surgery [1]. Congenital dysfunction of the ophthalmic branch of the trigeminal nerve represents an additional cause of corneal anesthesia, often treatable with recent advancements in corneal neurotization procedures [19,20]. Iatrogenic etiologies of corneal anesthesia are diverse, including damage from refractive surgeries such as laser-assisted in situ keratomileusis (LASIK), neurosurgical procedures, and some topical medications prescribed for other ocular conditions [21,22].

Systemic disease states that have been reported to impact the ophthalmic nerve and corneal innervation are numerous and include acquired neuropathies—such as diabetic and chemotherapy-induced—as well as inherited neuropathies such as Charcot-Marie-Tooth disease [2,23]. NK has rarely even been reported in association with uncommon disease states such as leprosy, as well as metabolic etiologies such as vitamin B-12 deficiency [24,25].

Outside of overt pathology, unique patient characteristics such as age and eye color have also been theorized to impact corneal sensation, though these are frequently debated. The use of contact lenses and increasing age have at times been found to be associated with loss of corneal sensitivity by some groups, though others have found no significant association [3,26,27]. Lighter pigmented irises have been shown to be associated with decreased sensation in some studies [26,27,28,29]; however, other studies have found increased corneal sensitivity, no significant association with sensation, or significant increases in exclusively chemical sensitivity [26,30,31]. In general, many benign patient characteristics have been studied over the years, and while findings have varied greatly, certain protective and predisposing characteristics are commonly suggested and explored. As studies employing more than one type of aesthesiometer occasionally only find differences in corneal sensation thresholds by the use of one device [3], the increased precision and accuracy of novel aesthesiometers may allow for better differentiation of these characteristics. The development of more readily available and easily comparable esthesiometers may also help elucidate additional characteristics that predispose patients to the development of corneal hypoesthesia and its complications.

## 4. History of Corneal Aesthesiometry and Its Advancements

Due to its simplicity and availability in most clinical settings, the cotton wisp test is the most commonly used qualitative assessment of corneal sensation. A cotton wisp is lightly pressed on the cornea to elicit a blink or slight irritation, which indicates an intact ophthalmic nerve branch [32].

Corneal sensation first became quantifiable in 1894 when von Frey attached varying lengths of horsehair to glass rods in order to test for abnormal corneal tactile sensation [15]. Because longer hairs were more likely to bend when used to apply pressure to the cornea, patients who were able to feel longer hairs were deemed to have greater corneal sensitivity, whereas those requiring shorter lengths to report sensation had lower corneal sensitivity. Boberg-Ans later improved upon von Frey’s method in 1955 by replacing horsehair fibers with a nylon filament. In 1966, Cochet and Bonnett improved upon the holder of the aesthesiometer filament by making it more portable, and later expanded the available widths of the retractable nylon filament to 0.08 mm and 0.12 mm [33]. The current Cochet–Bonnet aesthesiometer filament length can be adjusted between lengths of 5 and 60 mm, allowing the pressure exerted on the cornea to range from 11 to 200 mg per 0.0113 mm^2^. This is the current gold standard for obtaining a quantitative measurement of corneal sensation [27].

Further advancement of aesthesiometry tools took place in 1999 with Carlos Belmonte’s development of the Belmonte Noncontact Aesthesiometer [17]. Using a gas jet of carbon dioxide of adjustable flow and temperature, this device allowed for corneal stimulation by thermal, mechanical, and chemical means [17,34]. Later development of the CRCERT-Belmonte aesthesiometer allowed the clinician to control the temperature of the gas jet to minimize the thermal component of the stimulus [35]. The next innovation in esthesiometry came as a further modified noncontact aesthesiometer—the Swiss Liquid Jet Aesthesiometer—which employed small saline droplets at varying pressure levels to detect evidence of corneal hypo- or anesthesia [35,36]. Most recently, the introduction of the Corneal Esthesiometer Brill, which employs a pulse of air to the corneal surface, offers a handheld alternative to traditional non-contact corneal aesthesiometers [37] (Table 2).

Within the cornea, sensation thresholds vary by region, frequently delineated as the central cornea and four peripheral quadrants: superior, inferior, nasal, and temporal. Since the early days of measurement, the central cornea has been recognized as the most sensitive region, with the superior cornea as the least sensitive of the peripheral quadrants, theorized to be due to being in frequent contact with or covered by the upper eyelid [38,39,40]. With the introduction of devices with varied stimuli, specifically the pneumatic stimuli achieved by gas-based aesthesiometers, variation in sensitivity across corneal regions has been found to be less prominent in some studies. For instance, in 2007, Situ et al. reported that pneumatic cool and mechanical stimuli responses varied only slightly between the central cornea and two measured peripheral regions, temporal and nasal, and chemical sensitivity was measured to be approximately constant [41].

**Table 2:** Selected characteristics of available aesthesiometers.

Technique	Timeline	Advantages	Disadvantages	Force Parameter Measured	Reported Force Exertion Range
Cotton Wisp Test	-	- Simple and readily available - Inexpensive	- Highly subjective - Not quantifiable	N/A	N/A
Cochet–Bonnet Aesthesiometer	1966	- Widely used, commercially available device - Simple design - Portable	- Subjectivity and environmental factors impact repeatability [42] - Limited range of forces not suitable for evaluation of low corneal sensitivity [35,43,44] - Risk of injury to corneal epithelium [42,45]	Nylon filament length, correlated to manufacturer-provided force	0.06–2.56 mN [46]
Belmonte/Non-Contact Corneal Aesthesiometer	1999	- Mechanical, chemical, and thermal stimuli [47] - Greater sensitivity than Cochet–Bonnet [46] - Non-invasive [48] - Minimal discomfort to patients	- Complex operation limited to slit-lamp [34] - Not commercially available - Relatively expensive to implement	Air jet flow rate in milliliters per minute	<0.02–0.47 mN [46]
Swiss Liquid Jet Aesthesiometer	2018	- Allows for control of chemical and thermal stimuli [49]	- Complex operation limited to slit lamp [49] - Not commercially available - Relatively expensive to implement	Pressure of saline jet required to generate response	100–1500 mbar [49]
Corneal Esthesiometer Brill	2023	- Non-invasive - Minimal discomfort to patients - Handheld operation	- Relatively little comparative research available at this time	Pressure of air pulse in mbar	2–10 mbar [50]

Notable characteristics of tools commonly employed in the assessment of corneal sensation clinically, or in the study of corneal sensation.

## 5. Currently Described Instruments to Measure Corneal Sensation

### 5.1. Cochet–Bonnet Aesthesiometer

#### 5.1.1. Advantages

The Cochet–Bonnet aesthesiometer is a handheld device housing a retractable nylon filament in a pen-like shell. It is currently the most widely used aesthesiometer and is commonly cited as the gold standard tool for assessing corneal sensation and innervation. To obtain a measurement, the filament is first extended and applied to the cornea at its greatest length until a 5° bend is observed or the patient reports feeling the physical stimulus. If the stimulus is not registered by the patient, the nylon filament length is decreased in a stepwise manner until sensation is reported. This centimeter length is then correlated to a range of forces provided by the manufacturer to determine the force required for a response [33]. The shorter the length of the filament, the more force is required to bend the filament, resulting in a greater pressure exerted by the tool. Therefore, patients reporting sensation only at shorter filament lengths exhibit decreased corneal sensation and, subsequently, innervation [16,51]. This handheld device is the clinical standard for quantitative measurement of corneal sensation due to its portability, quantifiable measurements, and accessibility through various manufacturers. When it is used in a consistent manner by a trained clinician, its results can be informative in monitoring for improvement or worsening of corneal sensation within the limits of its measurement range.

#### 5.1.2. Drawbacks

While it remains the gold standard, one major drawback of the Cochet–Bonnet aesthesiometer is its reliance on contact of the filament tip with the fragile corneal epithelium during threshold measurements, which has the potential to cause injury [42,45]. Given that the measurement of corneal sensation often takes place in the context of existing corneal disease, the use of a stimulus with even minimal risk of damage to a weakened corneal epithelium is of concern in aesthesiometry [34].

Though intuitive in operation, Cochet–Bonnet aesthesiometers also vary in a user-dependent manner. Variations in the corneal location assessed, the subjective nature of what constitutes a bent filament, and the angle at which a clinician holds the aesthesiometer when approaching the eye can affect what is considered above or below threshold [42]. Additionally, the degree of apprehension the patient has regarding the invasive test and the visibility of the approaching instrument serve as other subjective factors with the potential to alter threshold values with the Cochet–Bonnet device [35,43,52]. This is especially true in the case of low-intensity stimuli, which the Cochet–Bonnet has been shown to be less effective at evaluating compared to other aesthesiometry devices [35,43,44]. This may be due to an inherent bend that develops when the nylon filament is extended at greater lengths, along with the limitation that the instrument’s lowest possible stimulus is often suprathreshold for many patients [35,43,46]. In these cases, more force is being applied than is clinically necessary for measurement, exposing these patients to greater risk of corneal trauma. Given that the instrument only allows for measurement in mm of nylon increments, the device may be less reliable at detecting small changes in sensitivity that may be clinically relevant to tracking a patient’s innervation status over time. This limitation may explain recent studies comparing corneal sensitivity between groups of patients where other aesthesiometers found statistically significant differences in response, while the Cochet–Bonnet did not [3]. The precision required for accurate measurements in addition to its subjectivity further limits the reliability of the Cochet–Bonnet in children.

Additionally, Cochet–Bonnet aesthesiometers are susceptible to variations in environment and time, as changes in ambient humidity and prior use of the nylon filament will cause variations in how easily the filament bends, and subsequently, the measurement of the force exerted [34,42]. Though it remains the most affordable device, replacement nylon filaments alone for the Cochet–Bonnet aesthesiometer can cost hundreds of dollars.

Finally, Cochet–Bonnet aesthesiometers measure sensory function using purely mechanical stimuli, differing from later aesthesiometers, which can evoke a response to chemical or thermal stimuli. While the clinical relevance of this limitation is yet unclear, it remains possible that the regular assessment of different types of nerves could result in improved understanding and diagnosis of a variety of complex ocular disorders resulting in NK which, to date, remain mechanistically unclear. Despite being comparatively affordable to implement relative to other aesthesiometry devices, Cochet–Bonnet aesthesiometers are still inaccessible to some due to cost. Additionally, the nylon monofilaments can be difficult or inconvenient to replace, and in some settings, are unable to be properly sterilized in accordance with standard facility and manufacturer policies. Because of this, cleaning between patients can be complicated, further increasing the risk for contamination and infection if not carried out properly. For example, the manufacturer states that the devices should be cleaned using glutaraldehyde disinfection, which is not available in most clinical settings, and the manufacturer does not recommend using ethanol for disinfection.

### 5.2. Gas-Based Non-Contact Corneal Aesthesiometers (NCCA)

#### 5.2.1. Advantages

The Belmonte Non-Contact Corneal Aesthesiometer (NCCA) measures corneal sensation by applying gas to the cornea, resulting in chemical, thermal, and mechanical stimulation. By doing so, the device allows for stimulation of the mechanosensory receptors seen in Cochet–Bonnet aesthesiometry, the less abundant cold receptors, and the more prevalent polymodal nociceptors that additionally respond to temperature extremes, irritants, and endogenous inflammatory mediators [47]. In an experimental study comparing this aesthesiometer to the Cochet–Bonnet, sensitivity was found to be higher in the Belmonte NCCA than the Cochet–Bonnet aesthesiometer [46]. Additionally, the NCCA offers a wider range of non-suprathreshold stimuli, allowing it to detect precise differences at lower levels of stimulation compared to the Cochet–Bonnet. The NCCA has better reproducibility, provides more information about a patient’s corneal sensation, is less subjective when compared to the Cochet–Bonnet, and employs more complex stimuli by activating mechanical, chemical, and thermal receptors of the cornea. Importantly, the Belmonte is non-invasive and can be safely used in the post-operative period due to the minimal risk of damage to the cornea [48].

#### 5.2.2. Drawbacks

One major limitation of the gas-based NCCA is the requirement of specialized equipment that lacks portability in comparison to the pocket-sized and portable Cochet–Bonnet. The instrument was briefly commercially available; however, it is now only used as a research tool as an alternative to the Cochet–Bonnet, rather than in a clinical setting. The most common method of use involves attachment to a slit lamp apparatus, making assessment outside of a devoted ophthalmology clinic setting where it is implemented difficult. Though it allows for more customization of the stimulus applied and, therefore, more detailed information to be collected outside of mechanoreceptor function alone, this comes at the cost of portability and accessibility relative to the gold standard Cochet–Bonnet. Additionally, gas-based NCCAs that are not able to control the variety of characteristics of the air jet present the challenge of determining what stimulus is being measured. This is because the CO_2_ gas used can cause local pH changes and a subsequent chemical stimulus that is irritating to the cornea, and evaporation caused by the jet of air can generate responses due to a cooling effect and/or depletion of the tear film [34]. The Belmonte NCCA additionally stimulates cold fibers, which may be more sensitive than other nerve fibers and thus estimate a higher level of corneal sensitivity than may be present [35]. This could, in theory, result in a patient with minimal corneal sensation to other stimuli being deemed to have normal sensation, though without clinical implementation, this remains unknown. Updated versions of the Belmonte Aesthesiometer combat these concerns by allowing for temperature control of the gas jet, though flow-rate-dependent evaporation and cooling remain issues [35]. Finally, the jet of gas produced affects a wider area of the cornea compared to contact-based aesthesiometry, which applies force over a single, small area in contact with the 0.12 mm filament [53]. As sensory fields of the cornea overlap considerably, this potential stimulation of multiple sensory receptors at once may limit the assessment of sensation in specific regions of the cornea.

### 5.3. Swiss Liquid Jet Aesthesiometer for Corneal Sensation (SLACS) 

#### 5.3.1. Advantages

The Swiss Liquid Jet Aesthesiometer for Corneal Sensation (SLACS) employs droplets of isotonic saline solution from a microvalve placed on a slit lamp with a temperature sensor, which allows this tool to match the ocular surface temperature to elicit a mechanoreceptor response [49]. The isotonic saline is not irritative to the cornea, in contrast to the CO_2_ gas employed by the Belmonte NCCA. In early variations of the device, developed by Ehrmann et al., the frequency of microvalve opening at a fixed pressure of 300 mbar controlled the stimulus strength, and was correlated with a patient’s degree of corneal anesthesia [36]. A modified version of this liquid jet aesthesiometer has since been adapted to apply differing pressure levels at a constant duration of 40 ms to control stimulus intensity [49]. This method of testing offers a large pressure range of 100–1500 mbar and a precision of 1 mbar. The intensity of the stimulus can be controlled to elicit a more precise response, allowing it to detect smaller changes in sensitivity relative to the Cochet–Bonnet. The liquid jet can also be controlled to match the surface temperature and chemical composition of the cornea, or differ in temperature and pH to assess isolated thermal and chemical sensitivity [49]. Compared to the Belmonte NCCA, the Swiss liquid jet does not cause secondary evaporative cooling, limiting confounding variables in measurement. Studies to date have suggested the SLACS to have reproducible results with precise localization to the ocular surface, allowing for the collection of more detailed information about corneal innervation in particular regions of the cornea [35].

#### 5.3.2. Drawbacks

The SLACS eliminates many of the remaining issues associated with gas-based aesthesiometers, though it shares some others. Like the gas-based systems, it is also not yet commercially available, and as such, it still warrants further evaluation before clinical implementation. The liquid jet system in its currently described form, compared to the Cochet–Bonnet, lacks portability, ease of use, and accessibility in many clinical scenarios due to also being an attachment to a slit lamp apparatus. Therefore, similar to gas-based NCCAs, its use is largely limited to research environments, and potentially devoted to ophthalmology clinic settings. The SLACS stimulates a wider area of the cornea relative to the Cochet–Bonnet device, though studies to evaluate this effect have found that a pulse stimulus approach can make localization on the ocular surface more precise [49].

Like gas-based NCCAs, relative limitations exist in comparing SLACs to the Cochet–Bonnet aesthesiometer, specifically, determining what physical property is being measured by the application of a complex stimulus. Additionally, as the surface area of the applied force of the SLACS is unknown and may vary based on the pressure and speed of the released jet, the pressure actually exerted on the corneal surface by the device is difficult to calculate with the current characterization of the liquid jet properties [49].

### 5.4. Corneal Esthesiometer Brill (Brill Esthesiometer)

#### 5.4.1. Advantages

Approved by the FDA in 2023, the handheld Corneal Esthesiometer Brill (Brill esthesiometer) addresses portability concerns of other non-contact corneal aesthesiometers, allowing non-contact aesthesiometry to be performed away from the slit-lamp apparatus. The device is battery-powered, can be used as a slit-lamp attachment or handheld device, and delivers pulses of ambient air as a stimulus, similar to the previously developed Belmonte NCCA. A camera and small screen on the Brill esthesiometer allows a clinician to pinpoint the area of intended corneal stimulation, and the alignment of two converging LED lights projected on the cornea from the device allows a consistent distance to be maintained between measurements [54]. The device offers five levels of stimulation ranging from approximately 1 mbar to 10 mbar, with an internal sensor at its outlet nozzle to record the pressure delivered to the corneal surface [50,54]. Given the device’s recent introduction, comparative research against other aesthesiometers is ongoing; however, one study suggests the Brill esthesiometer to be a safe portable alternative to the gold standard Cochet–Bonnet aesthesiometer, with comparable range and suitable agreement with Cochet–Bonnet sensation assessment of healthy and dry eyes [37].

#### 5.4.2. Drawbacks

The recent introduction of the Brill esthesiometer limits what we know about the device’s limitations and drawbacks. It is unclear if the five stimulus application levels of the Brill esthesiometer will be sufficient for monitoring relatively small changes in corneal sensation over time, such as those taking place over short periods of time in NK progression or resolution postoperatively. Additionally, it is unclear if the device will face similar limitations of other gas-based aesthesiometers due to their shared mechanism of stimulus generation. These may include difficulty with localization of the comparatively larger stimulus to measure regional changes in sensation, as well as flow-rate-dependent evaporation, cooling, and tear film depletion. As such, further study is needed to better understand its capabilities relative to traditional, non-handheld, non-contact corneal aesthesiometers, as well as confirmatory results relative to the Cochet–Bonnet aesthesiometer in application to patient care and decision making.

## 6. Recent Advancements and Alternative Corneal Assessment Techniques

Numerous advancements have taken place in recent years in not only aesthesiometry, but more broadly, morphologic corneal nerve and ocular surface assessment. While corneal sensation remains the only measurable surrogate marker for assessing the functional status of corneal nerves, increased study and adoption of ocular imaging techniques in recent decades has enabled the use of in vivo confocal microscopy (IVCM) and optical coherence tomography (OCT) as diagnostic aids for ocular surface diseases. These techniques allow for morphologic assessment of the cornea and its underlying structures that traditional evaluation by sensation threshold measurement, slit lamp examination, and fluorescein staining cannot provide.

IVCM allows changes such as nerve sprouting, tortuosity, and decreased corneal nerve density to be quantified, which can aid in diagnosis and provide insight into the severity of NK. By tracking these parameters over time, clinicians are able to monitor the efficacy of treatments intended to spur nerve growth, such as topical neurotrophic factors, or promote reinnervation, such as corneal neurotization. Further studies are needed to better understand the quantitative relationship between IVCM-assessed corneal nerves and functional outcomes, but early reports suggest a correlation between reductions in corneal nerve density and decreased corneal sensation. Alterations in the sub-basal nerve plexus of the cornea have been observed in corneal pathologies such as dry eye disease, and have been associated with decreased sensation [16,22]. In small studies, IVCM evidence of gradual and sustained reinnervation after neurotization has been reported, coinciding with clinically relevant improvement of the trophic state and sensation of the treated cornea [20,55,56,57]. In a study of 18 patients undergoing CN focusing on IVCM assessment of corneal nerve branch density (CNBD) and corneal nerve fiber trunk density (CNFD), it was found that the recovery of sensation correlated most strongly with increased CNBD, and that some patients with evidence of CNFD improvement without CNBD improvement did not entirely recover. This suggests that among the many parameters quantifiable by IVCM, the degree of branching after nerve trunk recovery may be closely related to the recovery of corneal sensory function [58].

Optical coherence tomography allows for morphologic evaluation of the anterior segment of the eye, including the epithelium, basal membrane, and stroma of the cornea—rather than nerves—and is particularly useful in assessing the severity of epithelial defects and corneal ulcers in patients with ocular surface pathology, including those secondary to NK [12]. Though not distinctly related to the assessment of corneal sensation, the clinically useful information afforded by increased access to and adoption of OCT has prompted some to advocate for updates to the Mackie classification system traditionally used to stage NK to include both IVCM and OCT parameters provided by Mastropasqua et al. in 2019 [12].

## 7. Future Directions and Conclusions

Understanding corneal sensation is an imperative aspect of understanding overall eye health. There are many tools used to measure corneal sensation and innervation, including the Cochet–Bonnet aesthesiometer, The Belmonte Non-Contact Aesthesiometer, the Swiss Liquid Jet Aesthesiometer, and the recently developed Corneal Esthesiometer Brill. These tools are relatively expensive, often require expertise and specialist training to use, and in some cases, are not easily adaptable to or accessible in the clinical or hospital setting.

Altogether, the limitations unique to each aesthesiometer provide insight into where future innovations should be directed, and where currently prototypic aesthesiometers may be able to improve to allow for eventual clinical implementation. Until recently, available corneal aesthesiometers presented the choice between accessible and intuitive devices that lack precision and accuracy, or precise and accurate devices that require greater expertise and more complex equipment. The Brill esthesiometer device seems to have been designed with addressing these limitations in mind, and only time, proper implementation, and careful research into the device’s characteristics will tell if these existing issues are resolved.

Despite its many drawbacks, the Cochet–Bonnet device still maintains its status as the “gold-standard” in the field due to its portability, simplicity in use, and output of measurements that are easy to correlate clinically. The Cochet–Bonnet aesthesiometer is the only widely used, commercially available device, and compared to other aesthesiometer devices, is significantly less expensive to implement. As such, advancements in the design of novel methods of measurement should balance between greater accessibility, ease of use, and precision to accurately identify and treat corneal disease.

In patients suspected to have impaired corneal sensation, such as those with NK, intervention often requires specialized expertise and equipment to assess the cornea and other ocular surface structures. Future aesthesiometers should be ubiquitously and easily available to clinicians. Ophthalmologists and optometrists should have access to instruments that are affordable, easy to use, provide reliable measurements, and use an established standard. By encouraging the use of such devices, the accurate diagnosis of NK can be made earlier, thereby opening the door to preventing the otherwise inevitable deterioration of the ocular surface.

While the accuracy of aesthesiometry has improved greatly since its induction, further innovation is required to meet the accessibility needs of the fast-paced healthcare system to improve patient outcomes, increase diagnostic accuracy, and allow for earlier interventions through medical or surgical treatment of a variety of ocular conditions.

## Figures and Tables

**Table 1:** Common corneal hypoesthesia etiologies.

Etiology	Mechanism	Esthesiometric Manifestations
Herpetic infection	Infectious loss of trigeminal ganglion neurons coupled with epithelial cell injury	Patchy, variable distribution of hypoesthesia/anesthesia
CNS tumor and/or resection	Loss of central afferent tracts	Total anesthesia
Prior ocular surgery	Direct injury to ciliary axons on ocular surface	Partial or total anesthesia
Developmental hindbrain syndromes	Agenesis of central afferents	Usually total anesthesia; often bilateral
Metabolic and pharmacological etiologies (e.g., diabetes, chemotherapy)	Neuron impairment and axonal loss	Partial hypoesthesia, often bilateral

Common etiologies of corneal hypo- or anesthesia, their key pathologic mechanism, and expected clinical manifestations on examination by corneal aesthesiometry.
